# Role of locum GPs in antibiotic prescribing and stewardship: a mixed-methods study

**DOI:** 10.3399/BJGP.2021.0354

**Published:** 2022-01-11

**Authors:** Aleksandra J Borek, Koen B Pouwels, Oliver van Hecke, Julie V Robotham, Christopher C Butler, Sarah Tonkin-Crine

**Affiliations:** National Institute for Health Research (NIHR) academic clinical lecturer;; Health Economics Research Centre, Nuffield Department of Population Health, University of Oxford, Oxford.; National Institute for Health Research (NIHR) academic clinical lecturer;; Antimicrobial Resistance, National Infection Service, Public Health England, London.; Nuffield Department of Primary Care Health Sciences, University of Oxford, Oxford.; Nuffield Department of Primary Care Health Sciences, University of Oxford, Oxford.

**Keywords:** antibiotic prescribing, antimicrobial stewardship, general practice, mixed-methods, primary health care, qualitative

## Abstract

**Background:**

Most antibiotics are prescribed in primary care. Locum or sessional GPs (locums) are perceived as contributing to higher prescribing and may face barriers to engaging with antimicrobial stewardship (AMS).

**Aim:**

To identify how locums’ antibiotic prescribing compares with other general practice prescribers, and how they perceive their role in antibiotic prescribing and AMS.

**Design and setting:**

Mixed-methods study in primary care.

**Method:**

Data on antibiotic prescribing, diagnoses, and patient and prescriber characteristics were extracted from The Health Improvement Network database. A mixed-effects logistic model was used to compare locums’ and other prescribers’ antibiotic prescribing for conditions that do not usually benefit from antibiotics. Nineteen semi-structured telephone interviews were conducted with locums in England and analysed thematically.

**Results:**

Locums accounted for 11% of consultations analysed. They prescribed antibiotics more often than other GPs and nurse prescribers for acute cough, sore throat, asthma and chronic obstructive pulmonary disease exacerbations, and acute bronchitis. The number of patients receiving antibiotics for these conditions was 4% higher (on absolute scale) when consulting with locums compared with when they consulted with other GPs. Four themes capture the perceived influences on prescribing antibiotics and AMS: antibiotic prescribing as a complex but individual issue, nature and patterns of locum work, relationships between practices and locums, and professional isolation.

**Conclusion:**

Locums contribute to higher antibiotic prescribing compared with their peers. They experience challenges but also opportunities for contributing to AMS, which should be better addressed. With an increasing proportion of locums in general practice, they have an important role in antibiotic optimisation and AMS.

## INTRODUCTION

Overuse of antibiotics contributes to antimicrobial resistance, one of the most important and urgent public health threats.^1^^,^^2^ In the UK, most antibiotics are prescribed in general practice,^3^ often for self-limiting infections.^4^^–^^7^ In 2013, the UK published its first 5-year national action plan for tackling antimicrobial resistance, which included antimicrobial stewardship (AMS) programmes.^8^^,^^9^ Despite these initiatives, antibiotic prescribing patterns still vary considerably across areas and practices, and some remain high prescribers.^5^^,^^10^^–^^12^ In the authors’ previous research, clinical commissioning group and general practice professionals perceived a high turnover of locum GPs as contributing to higher antibiotic prescribing, and locums as more likely than other GPs to be higher prescribers; this was because practices with transient staff were perceived as having less ownership of prescribing and locum GPs were perceived as less accountable for prescribing, less engaged in AMS, less aware of local guidelines, and lacking continuity of care.^13^^,^^14^

Locum or sessional GPs are registered, licensed GPs who work in temporary positions — often for multiple organisations — covering short-term absences and vacancies.^15^ A growing proportion of GPs (36%, >21 300 in 2017) work as locums in the UK.^15^ The National Association of Sessional GPs (NASGP) estimated that locums consult with around 36 million patients every year.^16^ Unlike ‘regular’, practice-based (salaried, partner) GPs, locums face unique challenges of working across organisations with different systems, contexts, and teams, and with unfamiliar patients that they may not see again. The NASGP highlights the many challenges faced and skills required by locums, noting that locums are generally undervalued, lack support, and may be scapegoated.^16^ Studies support the widespread negative perceptions and experiences of employing and working as locums, which can have adverse implications for the professional identity, organisations, and patients.^17^^–^^19^ A recent narrative review on the quality and safety of locums across settings and countries suggested that the context of locums’ work may increase risks; however, only eight empirical studies were identified, which were methodologically poor.^20^

Although locums form a substantial proportion of the GP workforce, there is little research on locums and, to the authors’ knowledge, none focused specifically on the role of locums in AMS. This study aimed to assess locums’ role in prudent antibiotic prescribing and explore their perceptions of AMS, which could help identify opportunities for further optimisation of antibiotic prescribing. Two research questions were addressed:

1. How does locums’ antibiotic prescribing compare to other general practice prescribers?
2. How do locums experience and view antibiotic prescribing and AMS?

**Table:** How this fits in

Locum or sessional GPs (locums) constitute over one-third of GPs in the UK (36% in 2017) but the patterns of and influences on locums’ antibiotic prescribing have been unclear. This study showed that locums were more likely than other GPs and nurse prescribers to prescribe antibiotics for acute cough, sore throat, acute bronchitis, and asthma and chronic obstructive pulmonary disease exacerbations. It also identified factors related to locum work that pose challenges to locums’ prudent antibiotic prescribing and engagement with antimicrobial stewardship (AMS) efforts. More focus is needed to engage locums in AMS, and practices that employ locums need to better communicate with and support locums.

## METHOD

A sequential explanatory mixed-methods study design was used to address the two research questions.^21^ The quantitative and qualitative data were analysed separately and sequentially; quantitative analysis addressed research question one and qualitative analysis addressed question two. Stakeholders, including professionals (for example, GPs and health psychologists) and Patient and Public Involvement representatives provided advice and feedback on the study (for example, on design and interpretation).

### Quantitative methods

Data on oral antibiotic prescribing, diagnoses, and patient, practice, and prescriber characteristics were extracted from The Health Improvement Network (THIN) database, a primary care electronic database that is representative (∼7% coverage) of the UK population.^22^^,^^23^ The THIN database distinguishes antibiotic prescribing between different roles, including locums, salaried GPs, partners, and registrars. Data were included from English general practices that provided data for at least one full calendar year between 1 January 2013 and 31 December 2015.

The authors previously evaluated variation between practices in the percentage of patients being prescribed an antibiotic for common conditions that do not generally require antibiotics,^7^ and the extent of variation in practice-level antibiotic prescribing rates that can be explained by patient and practice characteristics.^10^ Using a similar approach, this study evaluated whether there were differences between the proportion of patients prescribed an antibiotic for common conditions when consulting with locums, compared with those consulting with other GPs and nurse prescribers. Prescribing patterns were not assessed for GP retainers, assistants, or other healthcare providers, owing to the low number of consultations associated with these groups.

The following conditions, for which antibiotics do not usually provide additional benefit, were included: acute bronchitis, acute cough, acute otitis media, acute rhinosinusitis, acute sore throat, asthma exacerbations, and mild acute exacerbations of chronic obstructive pulmonary disease (COPD). In line with previous work,^7^ patients with relevant comorbidities or complications and recurrent and chronic presentations were excluded to reduce potential confounding by patient frailty or severity of disease. Confidence intervals for the prescribing proportions were calculated using robust standard errors to take into account dependence between multiple episodes from the same patient/practice.

### Qualitative methods

Locums were recruited through newsletters of the Royal College of General Practitioners and NASGP. Those interested were emailed study information and asked questions to enable sampling. Participants were included if they primarily worked as locums in NHS general practices in England. They were purposefully sampled to ensure diverse characteristics (for example, time since qualification, patterns of working as a locum, and geographical area; see Table 1) and until saturation (considered as being when no new major findings related to the research question were identified in multiple sequential interviews).

**Table 1.** Interviewee characteristics (*N* = 19)

**Characteristic**	**Sample**
**Sex, *n***	
Male	12
Female	7

**Length of time since qualified as a GP (up to the time of the interview), range (median)**	2 months to 22 years (5 years)

**Length of time working as a locum, range (median)**	2 months to 10 years (3 years)

**Typical number of sessions per week worked as a locum, range (median)**	2–8 (5)

**Typical number of different practices per month worked in as a locum, range (median)**	2–8 (4)

**Other roles alongside locum work, *n***	13^a^
Salaried GPs	4
Extended hours or GP hubs	3
OOH, urgent care, or A&E	5
Academic, GP trainers, or examiners	8

**Primary medical qualification obtained outside the UK, *n***	3

**Training/experience before qualifying as GP, *n***	9

**Geographical areas of work, *n* ^b^**	
West Midlands	1
East of England	2
North West	2
London	3
South West	5
South East	8

**Number of CCG typically working in as a locum, *n***	
1	8
2	5
3	3
≥4	4^c^

a *The total in this row is higher than the number of interviewees because some had multiple roles.*

b *Number of locums exceeds 19 as some work across multiple areas.*

c *In London and South East. A&E = accident and emergency. CCG = clinical commissioning group. OOH = out of hours.*

Participants gave verbal consent to participate, which was recorded. Telephone interviews were conducted by a qualitative researcher and social scientist between November 2019 and February 2020 using a semi-structured topic guide (see Supplementary Appendix S1) exploring experiences of working as a locum, perceived influences on locums’ antibiotic prescribing and optimisation, awareness of and engagement with AMS initiatives, and suggestions for improvements. Participants were reimbursed for participation. Interviews were audiorecorded, transcribed verbatim, checked for accuracy, and anonymised.

Interviews were analysed thematically (taking an essentialist/realist epistemological stance),^24^ using NVivo (version 12) software. The first five transcripts were inductively and independently coded by three experienced qualitative researchers. The multiple coding^25^ was compared between the three authors and discussed to develop a coding framework, which was then used to analyse the remaining transcripts. New codes were added as necessary. Codes and categories were combined into themes to address the research questions.

## RESULTS

### Quantitative results

**Figure 1.**
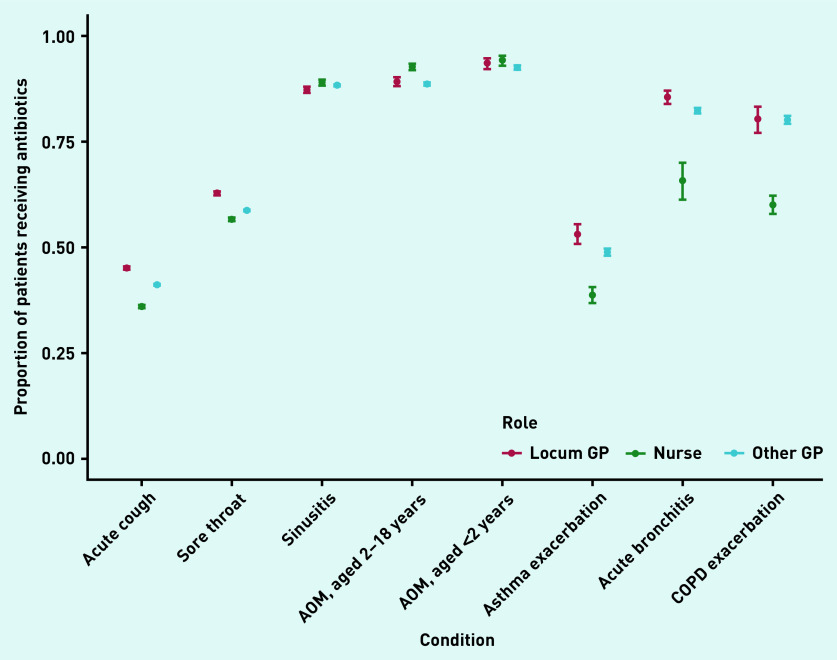
*Proportion of patients without relevant comorbidities receiving antibiotics when consulting with acute respiratory conditions with locum GPs, other GPs, or nurse prescribers in primary care. AOM = acute otitis media.*

**Figure 2.**
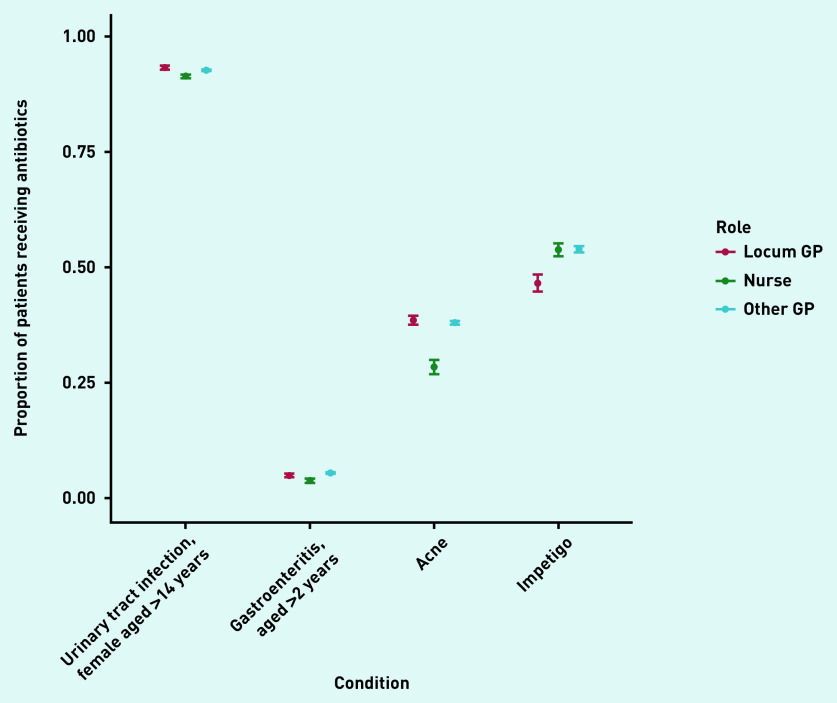
*Proportion of patients without relevant comorbidities receiving antibiotics when consulting with non-respiratory infections with locum GPs, other GPs, or nurse prescribers in primary care.*

In total, locums accounted for 11% of 1 511 787 consultations analysed. Acute sore throat (26% of all identified antibiotic prescriptions) and acute cough (38%) were the two main conditions for which antibiotics were prescribed (data not shown). The number of patients receiving antibiotics for these conditions was 4% higher (on an absolute scale) when consulting with locums compared with when consulting with other GPs (Figure 1). A similar difference was observed for asthma exacerbations and acute bronchitis, while prescribing percentages were more similar for other conditions (Figures 1 and 2). An exception was impetigo, for which other GPs prescribed antibiotics more often than locums (54%, 95% confidence interval [CI] = 53% to 55% versus 47%, 95% CI = 45% to 48%) (Figure 2).

For several conditions, nurses prescribed antibiotics in a smaller proportion of consultations (Figures 1 and 2) than locums and other GPs. However, this pattern was not consistent across all conditions: a higher percentage of children (aged 2–18 years) consulting with acute otitis media received antibiotics in consultations with nurse prescribers compared with locums or other GPs (Figure 1).

### Qualitative results

Nineteen locums were interviewed (Table 1). Interviews lasted 38–64 (mean 49) minutes. Four themes were identified to capture locums’ experiences and perceptions of influences on antibiotic prescribing and AMS. Findings are supported by quotes below, with additional quotes in Supplementary Box S1.

#### Theme 1. Antibiotic prescribing as a complex but individual issue

Interviewees described antibiotic prescribing as complex clinical decisions influenced by individual-level factors, such as the GP’s experience, skills, confidence, the patient’s clinical presentation (for example, symptoms), and expectations/behaviour. Some described an increasing awareness of AMS among GPs and patients; others argued that more change is still needed. They perceived locums as having an important role in AMS because they constitute many prescribers, but that role was seen as similar to all GPs’ responsibility for appropriate prescribing:

*‘It’s very much an individual GP responsibility really. If a practice stressed to me the importance of avoiding inappropriate antibiotics, I’d almost feel like they were stating the obvious* […] *all GPs should understand what appropriate antibiotic prescribing is, that’s real basic bread and butter general practice.’*

(Locum [L]1, male [M], 18 years since qualifying as a GP)

Participants also reported varied experiences and views of using AMS strategies (for example, guidelines, leaflets, delayed prescriptions, and clinical scores). Most did not know their individual antibiotic prescribing rates nor receive feedback on it, but they believed that they were low/prudent prescribers. They were interested in feedback on their prescribing and thought that it would be useful to locums and all GPs, and could be incorporated into GP appraisals. While most participants did not know the prescribing rates in practices they worked in, they had little or no interest in this, because as locums they described having no ‘vested interest’ in the practices:

*‘I’d be more interested in my own prescribing in comparison to the other doctors within the practice* […] *I don’t have a vested interest in any of the practices that I’m working in, it wouldn’t be that useful to me to know whether they’re high or low prescribers ...’*

(L9, M, 17 years)

However, they were also unsure how locums’ prescribing could be identified, as partner GPs’ names were often on prescriptions issued by locums.

#### Theme 2. Nature and patterns of locum work

The overall nature and patterns of locum work seemed to influence locums’ antibiotic prescribing. Participants worked across different practices and areas, which varied considerably in IT systems, workflows, and prescribing guidelines. This variation was challenging for locums who, as a result, might not always follow the local guidelines or workflows. For example, some participants reported following familiar prescribing guidelines from a different area to where they currently worked, and relied on IT prompts to indicate non-concordance with local guidelines. Participants suggested that working in one local area and regular, longer-term practices helped to minimise this challenge, and that adopting similar guidelines and approaches would make appropriate prescribing easier for locums.

Participants described how locums have more control and flexibility over their work. Thus, some requested longer appointments or catch-up slots to ensure sufficient time to provide good-quality care (for example, discussing antibiotics and safety-netting). Others perceived locums as being under more pressure from patients and time owing to hourly payment. This could lead to quickly ‘closing’ consultations with antibiotic prescriptions (rather than taking time to discuss patients’ perceptions about antibiotic treatment) to avoid running over:

*‘… the pressure that patients put on locums to prescribe, and the pressure of time on the locum* […] *it is to do with the time that we have, so you’re paid by the hour. You don’t necessarily want to run over* […] *think that makes a lot of locums more likely to not want to have that discussion to change patient perceptions about their use of antibiotics, and that it’s just easier to give antibiotics.’*

(L16, female [F], 4 years)

Participants also described another aspect of control and flexibility: being able to choose the practices where they work. For example, they reported avoiding practices perceived as being disorganised and struggling with demand and insufficient staff (and therefore having more staff turnover and less control over prescribing quality). They perceived appropriate prescribing and good-quality care as being more difficult in such practices.

Locums reported consulting more patients with acute infections who were more likely to require antibiotics (while patients with chronic illnesses were seen by regular doctors). They also reported that seeing unfamiliar patients and not being able to follow patients up can make locums more likely to prescribe antibiotics to avoid the risks of not prescribing, such as complaints and additional work for other GPs if patients re-consulted. However, participants also described how seeing unfamiliar patients may put locums in a better position to suggest a ‘new’ no-antibiotic approach, and that locums might be less concerned about potential negative impact of not prescribing on doctor–patient relationship:

*‘… because I’m not their normal GP* […] *I have the time and I have the fresh pair of eyes to go, “Actually, you know, things are changing a bit and I read something recently or I’ve been to an education session and how about trying without antibiotics this time?”’*

(L3, F, 22 years)

#### Theme 3. Relationships between practices and locums

Participants’ relationships and communication with practices varied considerably. They reported no communication about practices’ initiatives or approaches related to prescribing and AMS (for example, antibiotic-related targets and priorities), and said that they could participate or contribute if they were told about them (for example, during inductions). There was generally little to no feedback between practices and locums (unless concerning safety issues), and even less scrutiny over, and accountability for, locums’ prescribing than for regular clinicians. Participants reported that receiving feedback from practices would be helpful for them to improve and/or feel appreciated.

Participants described how locums were generally perceived, and felt to be ‘ *just to see patients*’ (L10, M, 3 years) and not a part of practice teams. Thus, some reported that locums have neither influence on, nor a role in influencing, practices’ antibiotic prescribing, or AMS initiatives:

*‘… you’re not part of the team and they don’t make you feel part of a team, you’re just there to come in and cover the session and that’s all you’re there to do. You’re not involved in discussions about prescribing or the processes in the practice* […] *it’s not really my role as a locum to get involved in trying to change processes that don’t seem to be working.’*

(L3, F, 22 years)

Others noted that locums working across many different practices have opportunities to observe, compare what works well with what does not, and identify potential improvements. Although locums’ feedback to practices was rare, some reported contributing to improvements in practices where they had good relationships.

Although participants reported that they practise similarly regardless of their role or where they work, the influence of different organisational cultures was apparent. Patient notes (that is, when and how consistently other GPs prescribed) and patients’ expectations for antibiotics gave locums a sense of the practices’ approaches to prescribing. Some reflected on being more inclined to prescribe antibiotics in higher-prescribing practices and where they would not feel supported by other GPs when not prescribing:

*‘I saw a patient today who has COPD and you look back and see that’s what they do and I’m more inclined to prescribe antibiotics just in case because that’s what they do, rather than making my own judgment, I’m just following what the practice are doing.’*

(L11, F, 5 years)

#### Theme 4. Professional isolation

Participants described GPs as *‘working in silos’* (L10, M, 3 years) and locums as even more professionally isolated than practice-based GPs. This was exacerbated by limited or no communication from commissioners, being less connected to professional groups and networks, and having to participate in professional training and meetings during unpaid time. Consequently, locums found keeping up to date with guidelines, evidence, and training more challenging, and having fewer opportunities for peer learning. This could contribute to less appropriate prescribing:

*‘* [Locums] *are a little bit outside* […] *the mainstream GPs who are going to all the regular CPDs* [continuing professional developments] *and GP updates and maybe are more aware of the problems around antibiotic stewardship and resistance* […] *as a locum you can go to no CPD meetings* […] *you can be far away from the nourishing flow of information* […] *and you can see how you can have a very different viewpoint about antibiotic prescribing.’*

(L10, M, 3 years)

Nevertheless, some participants described locums as well trained and aware of evidence as a result of being more proactive about their professional development. Many reported being proactive about ensuring they had access to relevant resources (for example, bookmarking online guidelines/tools) and communicating with practices and peers (for example, asking about training opportunities and joining meetings) to provide good care.

Participants discussed how practices and commissioners should better integrate and support locums; for example, by circulating information, updates, and training opportunities to all GPs registered on Performers Lists. Some suggested that AMS-related training should be mandatory, and that local peer groups for locums should be encouraged. Finally, some suggested that locums should be better recognised as a considerable professional group, and involved in wider policy development:

*‘We’re a significant amount of the workforce and we prescribe lots of antibiotics* […] *However, because we can’t influence local policy, perhaps our involvement would be limited only based on our personal experience* […] *our voices are minimally heard in general when we talk about health policy, just because locums and sessional GPs is just this nebulous group that aren’t really organised very well. So I think you’re missing probably lots of people that could add value to any sort of policy discussion.’*

(L5, F, 7 years)

Box 1 summarises the main influences reported by the interviewees on locums’ antibiotic prescribing, and suggestions for potential improvements.

**Box 1.** Summary of perceived influences on locums’ antibiotic prescribing, strategies, and suggestions

**Challenges and reasons for higher antibiotic prescribing**	**Opportunities and reasons for lower antibiotic prescribing**	**Strategies used by locums to manage challenges**	**Suggestions**
See more acute patients See more unfamiliar patients with limited follow up (and wanting to avoid work for others) May feel less accountable for their prescribing (no audit or feedback) May feel less invested in or concerned by antibiotic prescribing in practices where they work as locums Less (access to) training and peer learning May be under more pressure from patients seeking antibiotics May feel under more time pressure (antibiotic prescribing is seen as quicker than not prescribing) Less aware of practices’ AMS initiatives May feel influenced by practices’ high-prescribing culture and feel unsupported when not prescribing antibiotics (want to avoid risks and complaints)	No pre-existing relationship and expectations from patients (easier to suggest a ‘new’ no-antibiotic approach and less worried about impact on the relationship) Well trained and aware of the evidence May work more flexibly and take longer in consultations if needed to provide good care	Use typical AMS strategies (for example, guidelines and clinical scores) Select practices that are ‘good’ to work in, and avoid practices perceived as more disorganised and with higher staff turnover Work locally and in regular, longer-term practices Ensure extra time to familiarise with new practices Keep own notes/information/links related to local guidelines, processes, and patients to follow up Agree/request sufficient time for good-quality care Initiate communication with colleagues and take time to develop good relationships Ask for support when needed Rely on IT prompts for first-line antibiotic Ask practices for information about relevant training or meetings and attend them Join local GP groups or locum organisations	Audit locums’ prescribing Enable locums to issue prescriptions signed with their names, and link locums’ prescribing to their roles Provide feedback to locums, especially on individual antibiotic prescribing; invite locums’ feedback/suggestions for improvements to practices Use appraisal/revalidation to influence antibiotic prescribing (for example, require antibiotic prescribing audit and training) Adopt similar IT systems, guidelines, and processes across regions Improve inductions, including information about practice’s AMS approach and support for prudent antibiotic prescribing Use IT prompts and solutions to promote appropriate prescribing Organise locum peer groups, or include locums in local GP groups Provide free access to and encourage participation in AMS training Need whole-system approach to AMS, including ‘educating patients’

*AMS = antimicrobial stewardship.*

## DISCUSSION

### Summary

The analysis of prescribing data showed that locums were more likely than other GPs and nurse prescribers to prescribe antibiotics for acute cough, sore throat, acute bronchitis, asthma, and COPD exacerbations. The interviews found that although locums perceived appropriate antibiotic prescribing and engagement with AMS as individual GP’s responsibility, they are nevertheless influenced by factors specific to, or reinforced by, the characteristics of locum work.

### Strengths and limitations

To the authors’ knowledge, this is the first study to identify the role of locums in antibiotic prescribing and stewardship in general practice in England. A mixed-methods design allowed two complementary questions to be answered: the in-depth qualitative data provided possible explanations for the quantitative data. Quantitative data were derived from a large database and used established analysis methods. Qualitative data involved a purposeful sample and reached saturation on the themes reported. Trustworthiness and credibility were ensured by involving multiple researchers in the analysis and interpretation, keeping of analytic notes, and sharing the findings/interpretations with participants, stakeholders, and the Patient and Public Involvement group. Relevant guidelines were followed for reporting of mixed-methods^26^ and qualitative^27^ studies (see Supplementary Tables S1 and S2 for checklists).

However, it is likely that a certain degree of misclassification affected the quantitative analyses. An unknown proportion of the consultations performed by locums could be incorrectly attributed to other GPs owing to the wrong identifier being used when entering the information about the consultation into the practice software. Given that default identifiers are more likely to be associated with individuals working in the practice, this may have resulted in a dilution bias reducing any difference between locums and other GPs. While the focus was on patients without relevant comorbidities or complications and excluded recurrent and chronic presentations, the possibility cannot be excluded that some of the (absence of) differences are explained by other differences in case-mix seen by nurse prescribers, locums, and other GPs. The quantitative data to which the authors had access dated from 2013 to 2015; prescribing patterns may have changed since then. It is possible that locums who volunteered to be interviewed had different views and experiences to non-volunteers. Finally, the findings are limited to locums practising in England, and their relevance to other parts of the UK may not necessarily be transferable.

### Comparison with existing literature

Although research on locums is limited, this study’s findings are generally in line with other studies. It was previously found that health professionals perceived locums as contributing to suboptimal antibiotic prescribing,^13^^,^^14^ and this study shows that locums may indeed be more likely to prescribe antibiotics for some conditions. A survey published in 2000 found that 31% of practices employing locums in England were dissatisfied with locums’ clinical competence and underprescribing or overprescribing.^19^ As in this study’s interviews, the literature points to some factors that may affect the quality of locum care and prescribing; for example, locums may be less aware of local policies, less familiar with the practice population, and less likely to participate in audits, professional development, and networks.^20^ Moreover, it was found that locums consider appropriate antibiotic prescribing a GP’s responsibility (regardless of their role as locums), but some may perceive no vested interest in knowing or improving antibiotic prescribing rates in the practices where they work as locums. Lack of continuity of care may also affect doctor–patient relationships, potentially creating barriers to prudent antibiotic prescribing.^28^ The present findings show some similarity to influences on antibiotic prescribing in out-of-hours (OOH) practice: lack of existing relationship with patients (with similarly mixed views on the direction of this influence), lack of follow up, changing workforce, and inconsistencies between prescribers.^29^ Strategies and resources to address such specific challenges seem to be lacking for locums as for OOH prescribers.^30^ Effective AMS interventions can benefit from being informed by qualitative studies that identify behavioural influences on antibiotic prescribing.^28^^,^^31^^–^^33^ For example, communication training to help address antibiotics without extending consultation time may help locums, similarly to all GPs^34^ and OOH prescribers.^35^ Interventions targeting subgroups of GPs (for example, early-career GPs) can also help.^36^ Thus, targeted interventions for locums to promote prudent antibiotic prescribing by addressing the identified influences may be beneficial.

This qualitative analysis identified many influences on locums’ antibiotic prescribing that related to interpersonal and organisational factors (for example, inadequate inductions, communication, and feedback), and contextual influences (for example, varied systems, guidelines, and professional isolation). Similarly, previous literature showed that most factors affecting the quality and safety of locum practice related to the organisational context and ways in which organisations deploy and support locums,^20^ highlighting that locums *‘can only work as well as their working environment’*.^16^ Moreover, it was found that locums may adjust their antibiotic prescribing to different practices where they work, influenced by different patient populations and prescribing cultures. An Australian study showed that while targeting antibiotic-related education and feedback at GP trainees may be helpful, inconsistencies in antibiotic prescribing among practice doctors contributed to internal conflicts for GP trainees, limiting the impact of the intervention.^36^ Similarly, other studies highlighted the role of organisational, social, and cultural contexts on antibiotic prescribing and AMS.^13^^,^^37^^–^^39^ Therefore, effective AMS interventions may benefit from targeting practice teams, and supporting better integration of locums into practice teams and wider professional networks.

It is also important to recognise that locums are a diverse group with varied working patterns and approaches, and not all factors are equally relevant to every locum. Moreover, the interviewees in this study described (developing) different ways for dealing with challenges and opportunities for locums to contribute to improving antibiotic prescribing and processes in practices where they work (for example, through being proactive, identifying what works well or could be improved, and suggesting ‘new’ no-antibiotic approaches to patients). The role and contributions of locums in patient care, and both challenges *and* opportunities for practices and locums need to be recognised.^16^ Importantly, these findings may also be relevant to other prescribing issues (for example, over-the-counter medications and opioids), and locum work in OOH services.

### Implications for research and practice

Suggestions for improving antibiotic prescribing are summarised in Box 1. Locums could be supported in more prudent antibiotic prescribing, for example: by improving inductions and communication about practices’ approaches to prudent antibiotic prescribing; auditing and providing feedback on locums’ antibiotic prescribing; providing (free) access to AMS training and/or making it mandatory; including locums in relevant updates and communication; and including antibiotic prescribing audits and training in GP appraisals. To enable accurate identification of, and audit and feedback on, locums’ prescribing, it is critical to ensure that prescriptions issued by locums include their names and can be linked in the clinical systems and databases to their roles. Moreover, managing variation in prescribing guidelines and clinical systems, enabling easy access to guidelines, and IT solutions may help locums working across practices and areas. Finally, future research and AMS interventions could target locums (to address specific professional issues) and practice teams for consistent, practice-based approaches to AMS.

To conclude, this study showed that locums may contribute to higher antibiotic prescribing and identified possible reasons. Locums face specific challenges to optimising antibiotic prescribing and engaging with AMS, including dealing with variation between practices and areas, more acutely unwell and unfamiliar patients, limited communication, feedback, and support, and professional isolation. They also have opportunities to contribute to AMS; for example, through more flexibility in consultations, ability to try ‘new’ no-antibiotic approaches with unfamiliar patients, and ability to identify and share good practice. These challenges need to be addressed and opportunities utilised. As locums are a considerable and growing part of the GP workforce their role in AMS and practices’ responsibilities towards enabling optimal prescribing by locums need to be recognised.
